# *In silico* assessment of a novel single-molecule protein fingerprinting method employing fragmentation and nanopore detection

**DOI:** 10.1016/j.isci.2021.103202

**Published:** 2021-10-01

**Authors:** Carlos de Lannoy, Florian Leonardus Rudolfus Lucas, Giovanni Maglia, Dick de Ridder

**Affiliations:** 1 Bioinformatics Group, Wageningen University, 6708PB Wageningen, The Netherlands; 2 Groningen Biomolecular Sciences & Biotechnology Institute, University of Groningen, 9747AG Groningen, The Netherlands

**Keywords:** Biocomputational method, Sequence analysis, Proteomics

## Abstract

The identification of proteins at the single-molecule level would open exciting new venues in biological research and disease diagnostics. Previously, we proposed a nanopore-based method for protein identification called chop-n-drop fingerprinting, in which the fragmentation pattern induced and measured by a proteasome-nanopore construct is used to identify single proteins. In the simulation study presented here, we show that 97.1% of human proteome constituents are uniquely identified under close to ideal measuring circumstances, using a simple alignment-based classification method. We show that our method is robust against experimental error, as 69.4% can still be identified if the resolution is twice as low as currently attainable, and 10% of proteasome restriction sites and protein fragments are randomly ignored. Based on these results and our experimental proof of concept, we argue that chop-n-drop fingerprinting has the potential to make cost-effective single-molecule protein identification feasible in the near future.

## Highlights

• A proteasome-nanopore construct can cleave a protein and interrogate the fragments
• In simulations, this process provides sufficient information to identify proteins
• An alignment-based classifier attains 97% accuracy on the entire human proteome

## Graphical abstract

**Figure:**
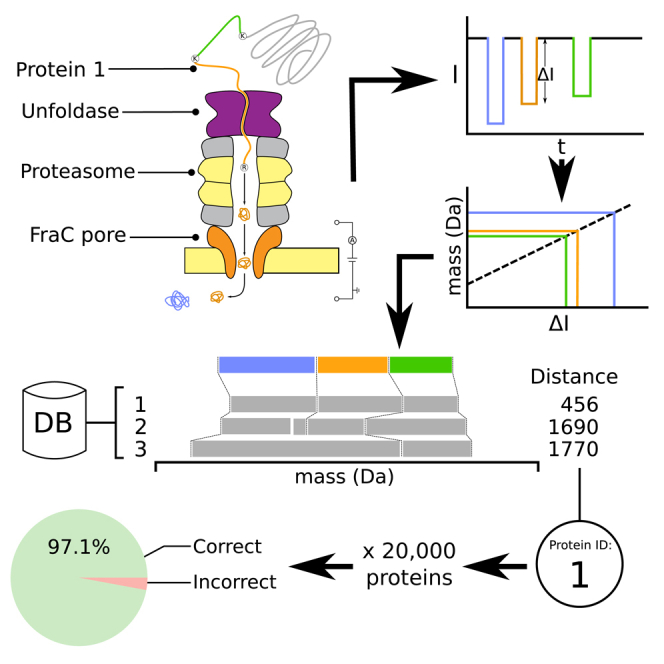

## Introduction

Over the past decades, mass spectrometry (MS) has allowed for ground-breaking discoveries in proteomics, enabling such impressive feats as the definition of a human protein atlas (Pontén et al., 2008) and large-scale screening for protein disease biomarkers (Keshishian et al. 2017). However, not all protein-related research questions may be addressed by MS. Examples are found in the nascent field of single-cell proteomics which, following the example of single-cell transcriptomics, is expected to give unprecedented insight into cell functioning and pathology (Vistain and Tay, 2021). While MS has already made strides in this field by enabling the detection of proteins present at thousands of copies per cell (Specht et al., 2021), some important and clinically relevant proteins such as signaling molecules and transcription factors are expected to be present in the range of dozens of copies (Righetti and Boschetti, 2013). The development of novel single-molecule protein identification methods is therefore necessary to unlock the true potential of single-cell proteomics.

In the search for single-molecule alternatives to MS, two main venues are currently being explored. On the one hand, conceptual methods utilizing the read-out of fluorescent dyes attached to a subset of residue types have shown promising results (Ohayon et al., 2019; Swaminathan et al., 2018; Yao et al., 2015). However, methods using fluorescence-based readout strategies require efficient and specific labeling of residues. Optimizing labeling strategies is non-trivial (e.g. Abello et al., 2007; Nanda and Lorsch, 2014), and less-than-perfect labeling may decrease accuracy; thus, a label-free method would be preferred.

On the other hand, unlabeled proteins may be analyzed using a nanopore, over which an electrical potential is applied; as a protein is passing through the pore, changes in electrical resistance may give information on the protein's properties (Hu et al., 2021). Proteins may be analyzed in their folded states (Houghtaling et al., 2019; Huang et al., 2017; Ouldali et al., 2020; Piguet et al., 2018; Sutherland et al., 2004) which is relatively straightforward but does not provide sufficient information to discriminate between similarly shaped and charged proteins. Furthermore, no single pore aperture size is suitable for proteome-wide analysis due to the wide variety of protein sizes found in nature (Brocchieri and Karlin, 2005). Alternatively, proteins may be unfolded and threaded single file through the pore using a molecular motor (Brinkerhoff et al., 2021; Nivala et al., 2014). This approach allows for finer interrogation of the residue sequence and may analyze proteins of any size using a single pore aperture size.

In prior work, we showed that engineered complexes of heptameric nanopores and proteasomes can be readily assembled without loss of proteasome activity or electrical conductance of the pore (Zhang et al., 2020). Furthermore, we have shown that residual current through FraC pores correlates well with the molecular weight of passing protein fragments in the 500–1600 Da range (Huang et al., 2019). Presumably, this is because weight is correlated to properties that directly influence residual current, such as fragment size, shape, and charge (Di Muccio et al., 2019; Houghtaling et al., 2019; Hu et al., 2018; Waduge et al., 2017; Wilson et al., 2019; Zernia et al., 2020). We thus proposed that proteasome-nanopore constructs can be used to identify proteins, in a conceptual method dubbed chop-n-drop fingerprinting (Zhang et al., 2020). An unknown protein can be processed terminal-to-terminal by the construct, cleaving it at proteasome target sites, after which the molecular weight of sequentially released fragments can be estimated based on the residual electrical current as they pass through the nanopore. The sequence of measured fragment weights can then serve as a characteristic signature—a fingerprint—of the protein. Once proven, this fingerprinting method can easily be implemented in a highly parallel fashion by adapting existing hardware that was developed for nucleic acid sequencing. Compared to both MS and existing fluorescence-based measurement equipment, this hardware is inexpensive and has a small benchtop footprint, thus opening up opportunities for field diagnosis and in-house analysis for even small laboratories. It is as of yet however unclear whether chop-n-drop fingerprints are sufficiently characteristic to identify a single protein in highly complex mixtures.

Here, we present a computational analysis of the chop-n-drop method, in which we show that simulated fingerprints of all proteins in the UniProt human proteome can be accurately classified using a simple alignment-based method. Considering these and previously published experimental results, we argue that chop-n-drop fingerprinting is a promising concept for cost-effective single-molecule protein identification.

## Results

### Simulation and classification method

**Figure 1:**
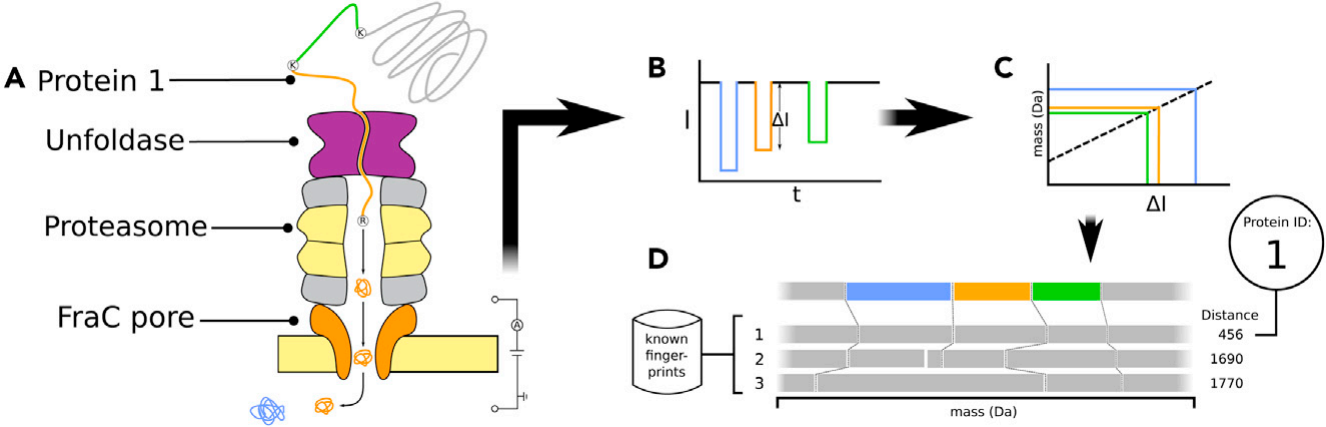
Schematic overview of the chop-n-drop fingerprinting method (A) A protein is unfolded by an unfoldase and fragmented by a proteasome directly introduced above a nanopore. The protease is engineered to lyse proteins at particular residues. (B) As the fragments pass the pore, a change in electrical current through the pore is measured. (C) The molecular weights of the fragments are estimated from the magnitudes of the current changes. (D) Finally, the produced sequence of fragment weights is aligned to database fingerprints of known proteins to identify the protein.

To estimate the performance of the chop-n-drop fingerprinting method on a highly complex protein identification task, we developed a simulation pipeline mimicking the experimental procedure, including several sources of biological and technical noise that we expect to encounter (Figure 1).

In essence, the chop-n-drop fingerprint of a protein only consists of a sequence of weights, which are deduced from pore current blockades caused by sequentially cleaved-off fragments passing through the nanopore. The simulation of this process follows a straightforward two-step process. First, akin to the proteasome cleaving a protein into fragments, we divide a given protein sequence into sub-sequences by splitting it at the proteasome's target sites. We assume here that we can force it to exhibit only trypsin-like behavior by mutating proteasome subunits exhibiting chymotrypsin-like and caspase-like activities, while leaving its subunits exhibiting trypsin-like activity intact (Collins et al., 2010; Li et al., 2004). To account for the fact that the proteasome will likely fail to cleave at a fraction of target sites, we only cleave each target site with a certain probability, which we refer to as the proteasome efficiency (*e*_*p*_).

Subsequently, we mimic the passing of fragments through a heptameric FraC pore, the readout of the current blockade, and the estimation of the fragment weight by simply translating the sub-sequences into corresponding fragment weights. Although weights can be calculated from sequences with high accuracy, experimental measurements may be less accurate and marked by a given resolution (*r*), the smallest detectable weight difference. In experimental setups, this parameter is dependent on pore and measuring equipment properties. The smallest weight difference we have detected with FraC so far is 4Da (Figure S1); thus, in simulations, we consider *r*-values above 4 Da attainable. To account for resolution in simulation, Gaussian noise is added to fragment weights, where the standard deviation of the noise is related to *r* (see STAR Methods). Fragments weighing less than 500 Da are removed, as they typically escape detection of heptameric FraC nanopores (Huang et al., 2019). Furthermore, as it has not been shown that the relation between weights above 1.6 kDa and current blockades remains monotonic (Huang et al., 2019), all fragment weights larger than this value are reduced to 1.6 kDa. Lastly, although we expect the seal between proteasome and pore to be extremely tight based on molecular dynamics simulations (Zhang et al., 2020), fragments may fail to enter the pore after cleavage. We account for this by only retaining each fragment with a certain probability, which we refer to as the capture rate (*C*). Although *C* is likely dependent on the size and charge of individual fragments, the relationship between these factors is unclear; thus, we assume *C* to be constant. The resulting sequence of fragment weights returned by this process constitutes the fingerprint for a protein.

We used fingerprints generated using our pipeline to develop a classification method, which assigns a protein identity to a given fingerprint. We follow an alignment-based approach, where a query fingerprint is aligned to a database of previously generated fingerprints, using a custom dynamic programming implementation (Figure S2). The database fingerprint that is most similar to the query fingerprint is assumed to have come from the same protein.

### Simulations under low-noise conditions

**Figure 2:**
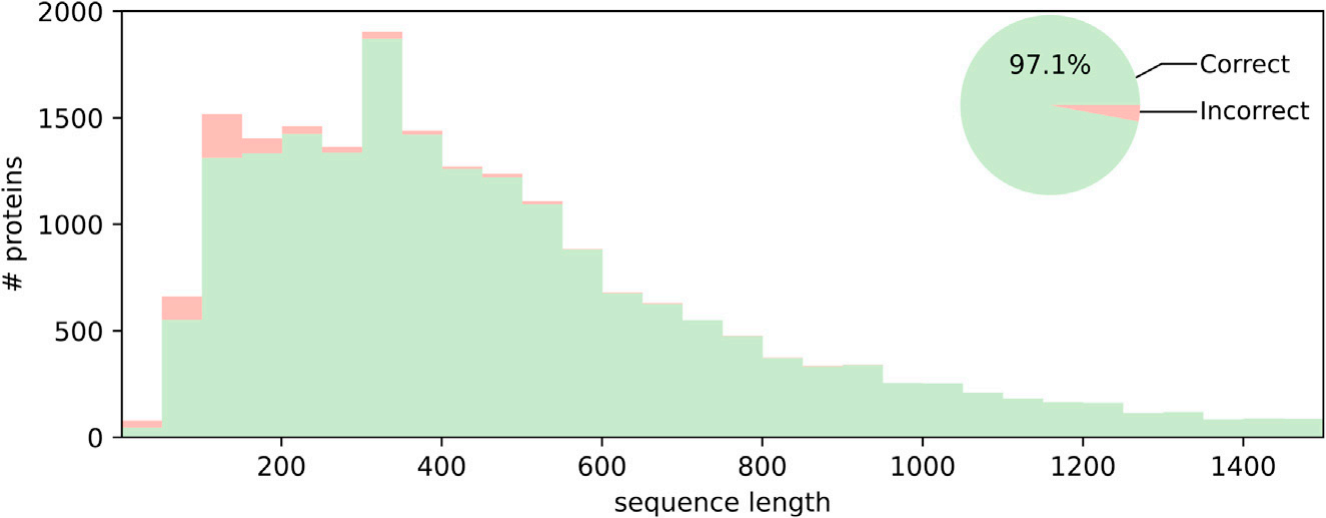
Simulated fingerprint identification accuracy assuming low-noise conditions Cumulative histogram of correct and incorrect classifications of simulated chop-n-drop protein fingerprints for all human proteome constituents, assuming low noise parameters; resolution *r* = 5 Da, capture rate *C* = 0.99 and proteasome cleaving efficiency *e*_*p*_ = 0.99. Numbers shown are distributed over sequence length (bars) and relative to the total number of proteins (pie chart). Alignment examples and sequence identity distribution for erroneous alignments are shown in Figures S3 and S4, respectively. Results assuming charge-dependent fragment capture are shown in Figure S5A.

We ran our simulation pipeline and classification method on all sequences in the UniProt human proteome (*n* = 20,395). Under close to ideal simulated noise parameters (*e*_*p*_ = 0.99, *r* = 5.0Da, *C* = 0.99), we find that our alignment-based approach retrieves the correct identity for 97.1% of fingerprints (Figure 2). Inspection of made alignments shows that our algorithm correctly handles missing and fused fragments (Figure S3A). Then, 77% of misclassifications occur for shorter proteins, under 250 residues in length. Of misclassified fingerprints, 42% shows more than 80% amino acid sequence identity to the protein as which it was wrongly identified, indicating that the resolution of 5Da assumed here is insufficient to consistently separate such similar entities (Figure S4). Upon inspection of these cases, we find that many misclassifications were in fact mix-ups between paralogous sequences. The remaining misclassifications are caused by chance alignments with different fingerprints (Figure S3B). This is expected to occur more often if a protein is shorter, as it will generally produce a fingerprint of fewer elements, which is less likely to yield a unique pattern.

### Simulations under high-noise conditions

**Figure 3:**
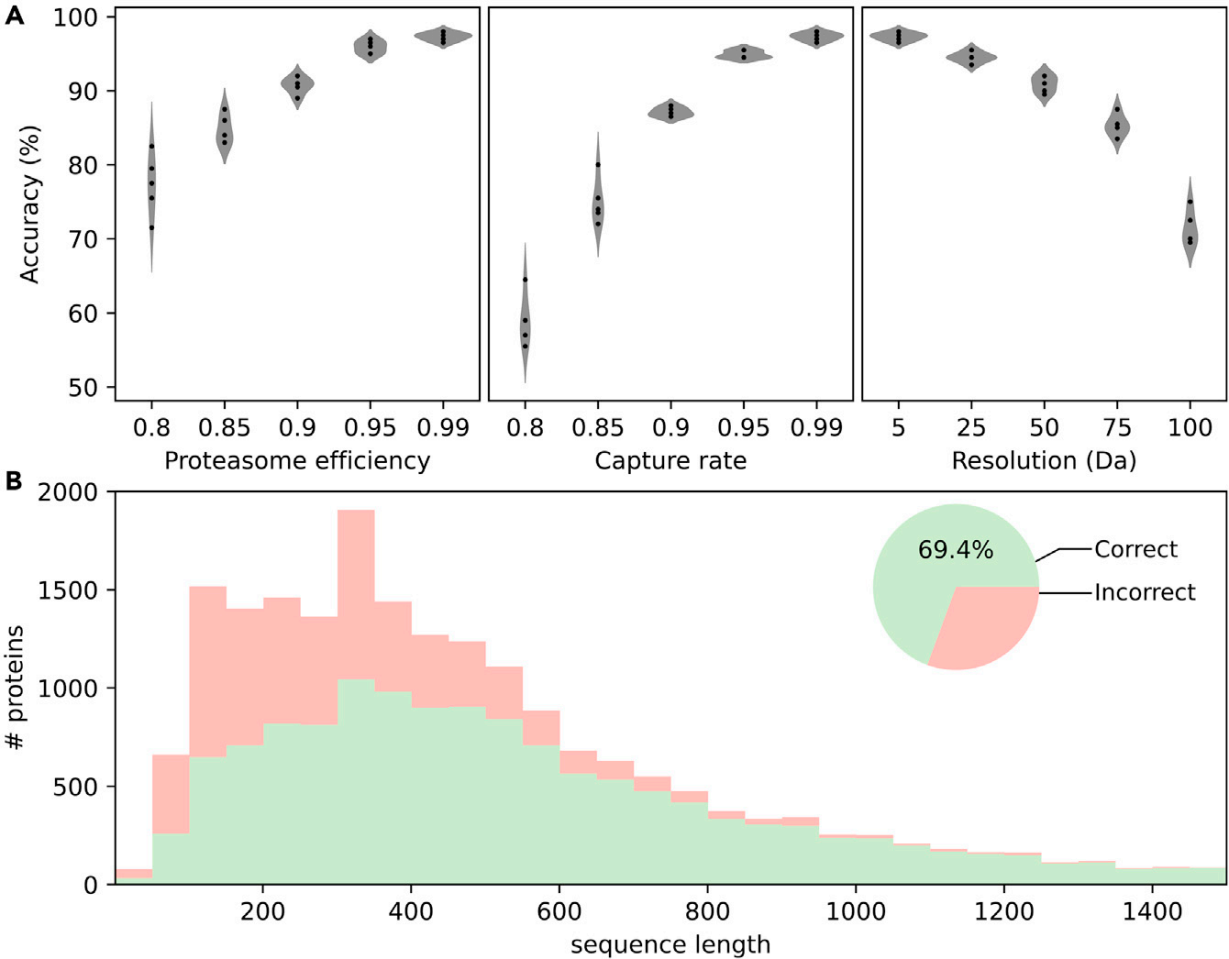
Simulated fingerprint identification accuracy under noisy conditions (A) Fingerprint classification accuracy over a range of noise parameter values; resolution (left), capture rate (mid), and proteasome efficiency (right). For each case the unvaried noise parameters are set to low-noise values (capture rate *C* = 0.99, resolution *r* = 5.0 Da, and proteasome efficiency *e*_*p*_ = 0.99). Five replicates were generated for each parameter combination. (B) Cumulative histogram of correct and incorrect classifications of simulated chop-n-drop protein fingerprints for all human proteome constituents, assuming more realistic noise parameters; *r* = 10 Da, *C* = 0.90 and *e*_*p*_ = 0.90. Numbers shown are distributed over sequence length (bars) and relative to the total number of proteins (pie chart). Results assuming charge-dependent fragment capture are shown in Figure S5B.

We subsequently probed how resistant chop-n-drop fingerprinting is to higher levels of experimental noise, by varying one noise parameter at a time while keeping all others near their low-noise values (*e*_*p*_ = 0.99, *r* = 5.0Da, *C* = 0.99). To keep computations tractable, these simulations were run on a random subset of 200 query sequences, while the reference database still contained all UniProt sequences so that the classification task was no less challenging. In each case, we find that accuracy deteriorates gracefully with parameter values (Figure 3A). Interestingly, we still attain an accuracy of 90.9% at a resolution of 50 Da, which is worse than the 44 Da resolution we reported previously (Huang et al., 2019) and more than tenfold worse than the current best resolution of 4Da, as reported in this work (Figure S1). Similarly, we find that a lower proteasome efficiency or capture rate of 90% still results in 90.7% and 87.1% accuracy on average, respectively. We then repeated the simulation on the entire dataset with all noise parameters at high-noise values (*e*_*p*_ = 0.90, *r* = 10.0Da, *C* = 0.90). Even under these circumstances, we find that 69.4% of proteins are correctly classified (Figure 3B). Here too, it should be noted that most incorrectly classified proteins were of lower sequence length.

Finally, we investigated the effect of fragment charge on accuracy. In FraC pores, electro-osmotic flow (EOF) is sufficiently strong to overcome an opposing electrophoretic force (EF) to some degree so that even negatively charged fragments are pulled through the pore toward an anode at the trans side. However, fragments carrying larger negative charges increase the EF so that the EOF can no longer cancel it out, and thus, such fragments cannot enter the pore. We investigated the effect of the omission of fragments that are too negatively charged (˂−1*e*) at FraC's operating pH levels (pH = 4.0) (Huang et al., 2019) and found that the effect on accuracy is negligible in both low-noise and high-noise scenarios (97.1% and 69.3%, respectively, Figure S5).

## Discussion

Single-molecule (SM) protein fingerprinting holds great promise to revolutionize biological research and diagnostics (Restrepo-Pérez et al., 2018). We have previously proposed that this may be accomplished using a proteasome-nanopore construct, which cleaves a target protein into fragments and subsequently reads out the fragment weights (Zhang et al., 2020). Here, we present simulation results indicating that the produced sequence of fragment weights contains sufficient information to identify a protein.

The hypothetical construct investigated in our simulations consists of a heptameric dihelical FraC pore, which we show to be well suited to detect differences between fragment weights at high resolution and a proteasome exhibiting trypsin-like cleaving activity. In prior work, we have shown that constructs of artificial heptameric beta-barrel PA-pores and proteasomes can be built without loss of function of either component. Given the structural similarity, we are confident that our hypothetical construct can be built in a similar way, by replacing PA-pore monomers with FraC monomers and by making use of engineered proteasomes (Collins et al., 2010; Li et al., 2004).

In the presented simulations, we included sources of noise that may hamper fingerprint measurements in practice. We assumed that the proteasome may not cleave each target site that weight measurements may be inaccurate up to a given weight resolution and that not all cleaved-off fragments may be caught in the nanopore. Assuming higher noise parameter settings—a fragment capture rate and proteome efficiency of 90%, with a measurement resolution of 10Da—for each of these noise sources, we find that overall accuracy remains sufficiently high at 69.4%. As accuracy increases with protein length, we find that chop-n-drop fingerprinting should be particularly suitable to identify larger proteins.

Over the past years, the obstacles on the road toward SM protein fingerprinting have been attacked vigorously from multiple angles, with several groups showing promising initial results and proofs of concept. While each proposed method has shown particular strengths, we argue that chop-n-drop combines several properties not found together in other methods. First, unlike fluorescence-based methods (Ohayon et al., 2019; Swaminathan et al., 2018; Yao et al., 2015), it does not require the implementation of any labeling chemistries as properties of the target protein are read out directly, thus evading issues with erroneous labeling and simplifying sample preparation. As a trade-off, fluorescence-based methods are more sensitive to differences between proteoforms as long as the difference involves the position or presence of a targeted residue type. As we show here that even at high resolution our method misclassifies proteins with high sequence similarity to other entries, it is likely that differences between highly similar proteoforms may also remain unnoticed.

Different methods based on the readout of folded proteins by electrical current blockage of a nanopore have been proposed as well (Huang et al., 2017; Piguet et al., 2018; Yusko et al., 2017). These were unable to analyze a wide range of protein sizes however; as the pore lumen needs to be of an appropriate volume for the analysis of a given protein size, a single nanopore is not able to detect minute differences in both small and large proteins. Here, this problem is mitigated by the fragmentation step.

Most importantly, however, the hardware required to implement chop-n-drop fingerprinting in a highly parallelized setting can be readily borrowed from commercial platforms for DNA sequencing using nanopores, which are inexpensive and have already been miniaturized to a handheld format. As such, we envision that our method could soon fill a niche that no other method currently can: that of small-scale, in-house single-molecule protein identification.

In conclusion, we provide evidence that chop-n-drop fingerprints can provide sufficient information to identify proteins in complex samples and present a suitable alignment-based classification method. Upon optimization of the fingerprinting procedure, we envision that our method may see practical implementation in the near future.

### Limitations of the study

Our simulation builds on the assumption that fragment weight is correlated to the residual current measured while the fragment passes the nanopore. Indeed, we have previously shown that this is the case for fragments weighing between 500 and 1600 Da (Huang et al., 2019). However, it should be noted that rather than the fragment's weight, its volume, shape, charge, hydrophobicity, and interactions with the pore interior directly influence residual current (Di Muccio et al., 2019; Houghtaling et al., 2019; Hu et al., 2018; Waduge et al., 2017; Wilson et al., 2019; Zernia et al., 2020). As these properties are more difficult to model and considering how they apparently correlate to weight sufficiently well to in turn correlate weight and residual current (Huang et al., 2019), we consider using weight in simulated fingerprints justifiable. Once the experimental methodology has been further developed and protein fingerprints can be measured more routinely, we can define the relation between these properties and the residual current in more detail to predict fingerprints in a more robust manner.

The existence of different proteoforms, which was not accounted for in this simulation, presents both an opportunity and a challenge to chop-n-drop fingerprinting. Through alternative splicing and post-translational modification (PTM), multiple proteoforms with different functions may be generated from the same gene (Aebersold et al., 2018). Depending on the spliceoform or the PTM types present, different proteoforms may generate distinct fingerprints. This allows their individual identification at SM resolution, which is an important potential application of SM analysis, but also adds tens of thousands of potential fingerprint patterns, which further complicates the task of fingerprint classification. A solution may be to fractionate samples prior to chop-n-drop analysis, after which each fraction may be analyzed using a dedicated classifier which only considers the proteoforms that could be present in a given fraction.

## STAR★Methods

### Key resources table

**Table:** 

REAGENT or RESOURCE	SOURCE	IDENTIFIER
**Bacterial and virus strains**		

electrocompetent E. cloni ® EXPRESS BL21 (DE3) strain cells	Lucigen	Cat#60300

**Chemicals, peptides, and recombinant proteins**		

His6-tagged G13F Fragaceatoxin C (FraC)	Lucas et al., 2021	N/A
[Met5]-Enkephalin	Sigma Aldrich	M6638, CAS: 82362-17-2
[d-Ala2][d-Leu5]-Enkephalin	Sigma Aldrich	E7131, CAS: 94825-57-7

**Deposited data**		

Electrophysiological traces and simulated fingerprints	This paper	DOI: 10.5281/zenodo.5116022
UniProt Human proteome UP000005640	UniProt	https://www.uniprot.org/proteomes/UP000005640

**Software and algorithms**		

Simulation and analysis code	This paper	DOI: 10.5281/zenodo.5116022

### Resource availability

#### Lead contact

Further information and requests for resources and reagents should be directed to and will be fulfilled by the lead contact, Carlos de Lannoy (carlos.delannoy@wur.nl).

#### Materials availability

• The amino acid sequence for His6-tagged G13F Fragaceatoxin C (FraC) is included in the method details section.
• Plasmid pT7-SC1 containing Fragaceatoxin C (FraC) with a modification of glycine 13 to phenylanlanine and a C-terminal His6-tag was obtained from an earlier study (Lucas et al., 2021).
• Other reagents are available without restrictions, from sources mentioned for each reagent.

### Method details

#### Fragaceatoxin C purification

Plasmid DNA containing the sequence for His6-tagged Fragaceatoxin C (FraC) with a phenylalanine modification on position G13 was transformed into electrocompetent E. cloni ®EXPRESS BL21 (DE3) strain cells (Lucigen) for protein expression. Transformed cells were plated on Lysogeny broth (LB) plates containing 100 μg/mL ampicillin and incubated overnight at 37°C. A single colony was cultured in LB medium containing 100 μg/mL ampicillin and finally protein expression was induced using 0.5 mM isopropyl-β-D-thiogalactopyranoside (IPTG). Bacteria were harvested, treated with 0.2 units/mL DNase I and 20 μg/μL lysozyme, and sonicated, after which the mixture was incubated with Ni-NTA beads. FraC monomers were washed from the beads using elution buffer containing 0.300M imidazole. Sphingomyelin (porcine, brain):1,2-Diphytanoyl-sn-glycero-3-phosphocholine (DPhPC) liposomes for FraC oligomerization were created by solubilizing equal weight parts sphingomyelin and DPhPC in a pentane-ethanol mixture and evaoporating the solvent to create a lipid film. The film was then solubilized in 0.015M Tris-HCL dilution buffer (pH 7.5, 0.150M NaCl), briefly sonicated, frozen at −20°C and thawed. Liposomes and FraC monomers were mixed at a mass ratio of 10:1 solubilized in 0.6% (w/v) N,N-Dimethyldodecylamine-N-oxide (LDAO) and diluted in 0.02% (w/v) Dodecyl-β-D-maltoside (DDM). Finally oligomerized FraC was bound to Ni-NTA beads and eluted in oligo elution buffer (0.200M Na_2_EDTA, pH 8.0, 0.02% (w/v) DDM). This protocol was previously described in (Mutter et al., 2021)

#### His6-tagged fragaceatoxin C sequence

MASADVAGAVIDFAGLGFDVLKTVLEALGNVKRKIAVGIDNESGKTWTAMNTYFRSGTSDIVLPHKVAHGKALLYNGQKNRGPVATGVVGVIAYSMSDGNTLAVLFSVPYDYNWYSNWWNVRVYKGQKRADQRMYEELYYHRSPFRGDNGWHSRGLGYGLKSRGFMNSSGHAILEIHVTKAGSAHHHHHH.

#### Electrophysiological recordings

For planar lipid bilayer electrophysiology measurements, a constant voltage of −70 mV was applied over a single nanopore, and a buffer solution containing 1M KCl buffered to pH 3.8 using 50mM Citric acid titrated with bis-tris-propane was used. [Met5]-Enkephalin and [d-Ala2][d-Leu5]-Enkephalin (Sigma Aldrich) were added in 10μM concentration. Ionic currents were recorded using an Axopatch 200B amplifier coupled with a Digidata 1440a or Digidata 1550B A/D converter (Molecular Devices). All data was recorded using Clampex 10 (Molecular Devices) with a sampling frequency of 50 kHz and an analog Bessel filter of 10 kHz. Measurement procedures were first described in (Lucas et al., 2021).

#### Electrophysiological recording analysis

Data was analyzed using Python 3.7 and is contained within a Jupyter notebook, available in the Github repository noted in the key resources table. Events from translocating peptides were characterized using a threshold search algorithm combined with a generalized flat-top normal distribution fit (Lucas et al., 2021). The resolution between the two peptides was determined using the difference between the peak centers and their standard deviation (Lucas et al., 2021).

#### *In silico* fingerprint generation

Code for *in silico* fingerprint generation and classification was written in Python 3.8 (Python Software Foundation, www.python.org). We generate *in silico* chop-n-drop fingerprints by splitting protein sequences at protease target sites and calculating the weights of the resulting fragments from their sequences. We assume that fragments of a weight lower than 500 Da are undetectable, thus these fragments are removed from fingerprints. Fragments of a weight larger than 1.6 kDa are set to 1.6kDa, as prior investigations showed that the relationship between weight and current blockage is not monotonic above this weight (Huang et al., 2019). In simulations where fragments were selected based on charge, the fragment charge was calculated using biopython (v1.78), which employs a concentration ratio-based calculation (Bjellqvist et al., 1993).

Three parameters are set to represent different noise sources; capture rate *C*, proteasome efficiency *e*_*p*_ and resolution *r*. The capture rate denotes the fraction of fragments that enters the pore after lysis and is measured. In our simulations each fragment is retained with a probability of *C*. The proteasome efficiency denotes the fraction of target sites at which the proteasome cleaves. In simulations, each target site has a probability of *e*_*p*_ of being cleaved. Note that a failure to cleave will result in two fragments being fused together, after which they remain represented in the fingerprint as the sum of their weights. Finally, the resolution denotes the minimum difference in fragment weight that can still be detected by current blockage, expressed in Da. We adhere to an experimentally found minimum resolution of 4Da (Figure S1). In our simulations, the resolution is represented by the magnitude of Gaussian noise added to fingerprint weights. Specifically, we define the standard deviation of the distribution from which a noise value *n* is drawn such, that the probability of a fragment size measurement deviating *r* or less from its actual size is fifty percent:

(Equation 1) P(n≤r)=0.5

The standard deviation enforcing this resolution *r*, $\sigma_{r}$, can be found using the Z-score formula, in which the Z-score is calculated using the inverse cumulative distribution function of the standard normal distribution, ${\text{Φ}}^{-1}$:

(Equation 2) $$\sigma_{r}=\frac{-r}{{\text{Φ}}^{-1}\left(0.5/2\right)}$$

As resolution is expressed as a positive number and Equation 2 considers the lower tail of a distribution centered at 0, the resolution is multiplied by −1.

#### Simulation and classification

We ran *in silico* digestions on all sequences in the UniProt human proteome (UP000005640). To compile a database of fingerprints with known identity, we first performed an *in silico* digestion under noiseless circumstances (i.e. *C* = 1.0, *e*_*p*_ = 1.0 and *r* = 0.0Da). Then we ran several subsequent digestions for a range of values for *C*, *e*_*p*_ and *r*. Fingerprints from these runs were classified by aligning them to database fingerprints obtained from noiseless digestions.

We gauge the similarity of query and database fingerprints by aligning them using a dynamic programming algorithm (Figure S2). The dynamic programming table is filled as follows:

(Equation 3) $$\begin{array}{l} S\left(i,j\right)=\text{min}\left\{ \begin{array}{l} \left|X_{i}-Y_{j}\right|+S\left(i-1,j-1\right)\\ \left|X_{i}+X_{i-1}-Y_{j}\right|+S\left(i-2,j-1\right)\;i\leq \left|X\right|,j\leq \left|Y\right|\\ \left|X_{i}-Y_{j}\right|+S\left(i-2,j-1\right)+G\\ \left|X_{i}-Y_{j}\right|+S\left(i-1,j-2\right)+G \end{array}\right. \end{array}$$

with the following conditions for edge cases to ensure that the alignment is global:

(Equation 4) $$\begin{array}{l} S\left(0,0\right)=0\\ S\left(i,0\right)=\infty \;\forall \;1\leq i\leq N_{X}\\ S\left(0,j\right)=\infty \;\forall \;1\leq j\leq N_{Y} \end{array}$$

Here S(i,j) is the distance between query and database fingerprints *X* and *Y* respectively, up to fragments *X*_*i*_ and *Y*_*j*_ and *G* is a gap penalty. *N*_*X*_ and *N*_*Y*_ are the numbers of fragments in *X* and *Y* respectively. At each step in the alignment one of three actions may be taken. First, a single fragment of each fingerprint may be aligned, in which case the absolute difference of their weights is added to the total score. Second, two fragments of *X* may be aligned to one fragment of *Y*, corresponding to a missed proteasome target site. This action increases the score by the difference between the summed weight of the former and the single weight of the latter. Third, a gap may be introduced in either *X* or *Y* at the cost of a penalty. The gap penalty *G* is dependent on the resolution used during digestion:

(Equation 5) $$G={\left(1.96\cdot \sigma_{r}\right)}^{2}+L$$

Here $\sigma_{r}$ is the resolution-dependent standard deviation of Gaussian noise added to fragment sizes during *in silico* digestion (Equation 2) and *L* is the lower detection limit (*L* = 500 Da). This means that introducing a gap is preferred over matching fragments if the difference between fragment weights exceeds the difference expected in 95 percent of correct matches. The addition of *L* is required to ensure that a match is still preferred if a normally undetected fragment (i.e. of which the weight is under *L*) is fused to another fragment due to a missed proteasome target site.

A query fingerprint is classified by aligning it to all fingerprints in the database and assigning it the identity of the database fingerprint to which the distance is smallest.

## Data Availability

All generated data and analysis code was deposited on github, at: https://github.com/cvdelannoy/chop_n_drop_simulation.• Ion current measurement data have been deposited at Github and are publicly available as of the date of publication. DOIs are listed in the key resource table. • All simulation and analysis code has been deposited at Github and is available as of the date of publication. DOIs are listed in the key resources table. • For fingerprinting simulations we used all available sequences in the UniProt human proteome (UP000005640). • Any additional information required to reanalyze the data reported in this paper is available from the lead contact upon request.
