# Supplementary material for: In silico assessment of a novel single-molecule protein fingerprinting method employing fragmentation and nanopore detection
Source: iScience. 2021 Oct 1;24(10):103202. doi: 10.1016/j.isci.2021.103202 (PMC8521182; doi:10.1016/j.isci.2021.103202)
Supplement: Document S1. Figures S1–S5 [file mmc1.pdf]

iScience, Volume *24*

Supplemental information

In silico assessment of a novel single-molecule protein fingerprinting method employing fragmentation and nanopore detection

Carlos de Lannoy, Florian Leonardus Rudolfus Lucas, Giovanni Maglia, and Dick de Ridder

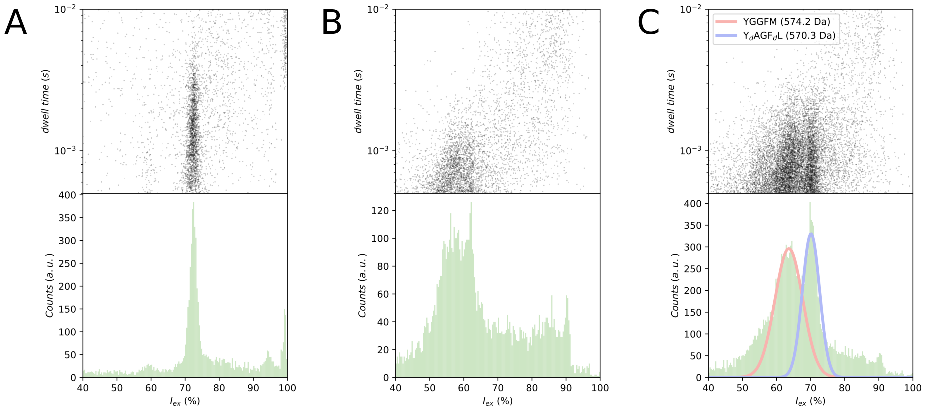

Figure S1: **Excluded current of two peptides with a difference in mass of 4 Da, related to STAR methods.** Top panels show excluded current (I_{ex}) set against the dwell time of events observed, bottom panels show the excluded current histogram of the top panel. Data is shown for **(A)** [Met5]-Enkephalin (YGGFM), **(B)** [d-Ala2][d-Leu5]-Enkephalin (YdAGFdL) and **(C)** an equimolar mixture of the two peptides, with fitted Gaussians denoting the peak for each peptide.

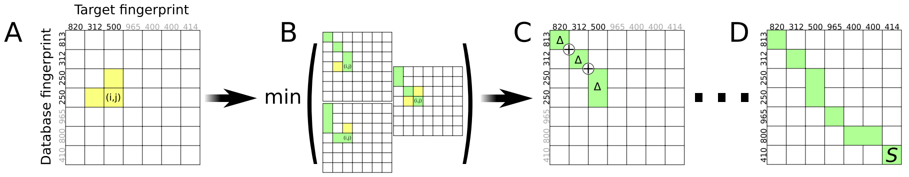

Figure S2: **Diagram of the dynamic programming algorithm used to align database and query chop-n-drop fingerprints, related to STAR methods. (A)** Numbers on the axes of the matrix denote fragment weights in Da. Weights involved in the displayed alignment step are black. The cells in the comparison matrix are filled row-wise starting from the top-left, by entering the alignment distance up to cell (i, j) in (i, j). **(B)** To fill square (i, j), two fragments of one fingerprint may be aligned to one fragment in the other, or single fragments may be aligned. A gap may also be introduced at a resolution-dependent penalty (not shown). **(C)** The option minimizing the summed distances of aligned fragments (Δ's) up to (i, j) is chosen – in this case, two fragments of the database fingerprint are aligned to a single fragment of the target fingerprint. **(D)** The process is continued until the bottom right cell is filled. The value in this cell (S) is the alignment distance for this pair of fingerprints.

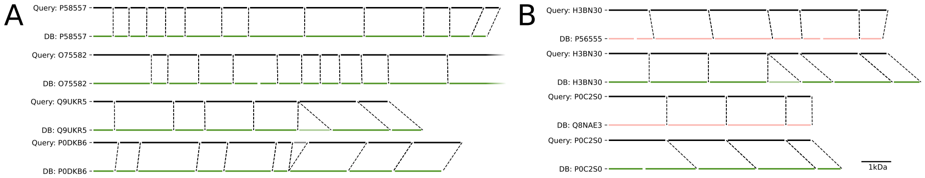

Figure S3: **Representative examples of alignments between chop-n-drop query fingerprints and the best-matching database fingerprints, related to Figure 2.** Dotted lines denote aligned fragments. Fragments for which a gap was introduced are greyed-out. **(A)** Four correct alignments. **(B)** Two incorrect alignments (red) and corresponding correct alignments (green).

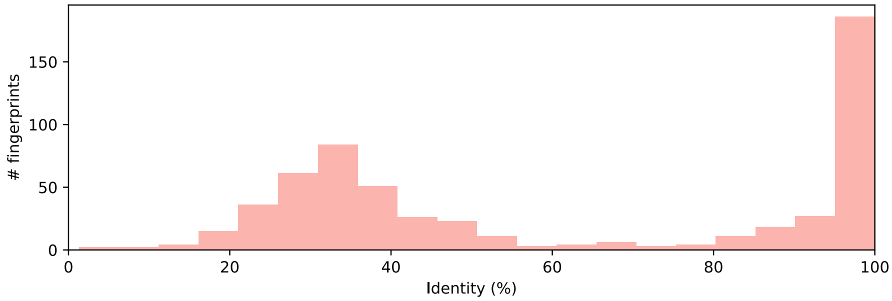

Figure S4: **Distribution of sequence identities between misclassified proteins and the proteins for which they were mistaken based on their chop-n-drop fingerprint, related to Figure 2.** Random alignments are expected to generate a sequence identity of thirty percent on average.

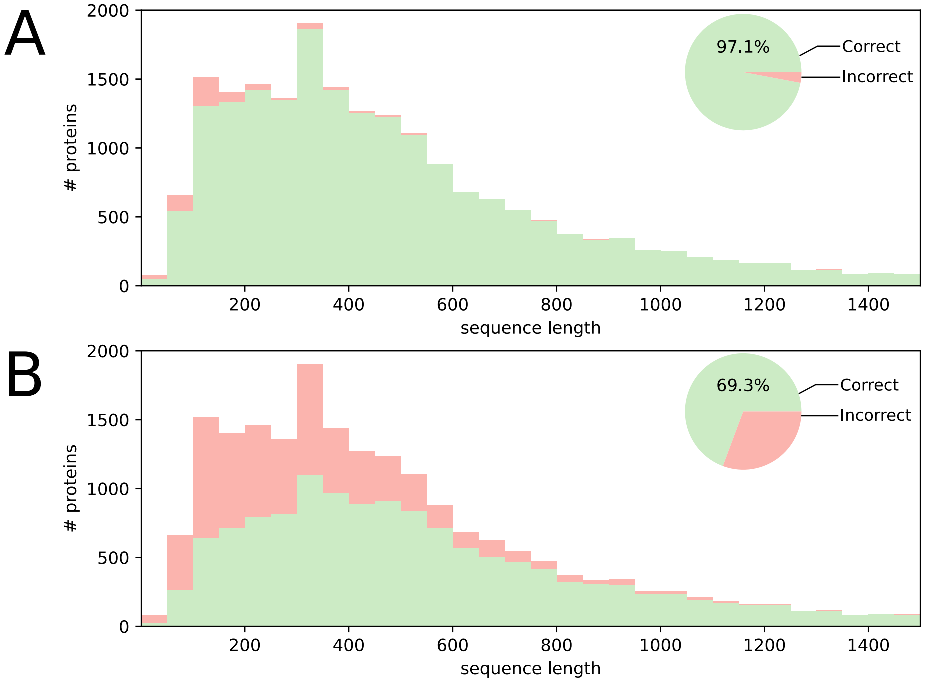

Figure S5: **Simulated fingerprint identification accuracy assuming charge-dependent fragment capture, related to Figures 2 and 3.** Cumulative histograms of correct and incorrect classifications of simulated chop-n-drop protein fingerprints for all human proteome constituents, accounting for fragment charge, related to Figures 2 and 3. Here it is assumed that fragments with a charge lower than -1 at pH=4 cannot pass the pore. Results are shown for **(A)** low-noise parameter settings (resolution r = 5Da, capture rate $C = 0.99$, proteasome efficiency $e_p = 0.99$) and **(B)** high-noise parameter settings (r = 10Da, $C = 0.99$, $e_p = 0.99$). Numbers are shown distributed over sequence length (bars), and relative to the total number of proteins (pie chart).
